# Pasireotide therapy in a rare and unusual case of plurihormonal pituitary macroadenoma

**DOI:** 10.1530/EDM-13-0026

**Published:** 2013-08-30

**Authors:** Rajesh Rajendran, Sarita Naik, Derek D Sandeman, Azraai B Nasruddin

**Affiliations:** Department of Diabetes and EndocrinologyThe Ipswich Hospital NHS TrustIpswich, IP4 5PDUK; 1 Department of Diabetes and EndocrinologyRoyal United Hospital Bath NHS TrustBath, BA1 3NGUK; 2 Department of Diabetes and EndocrinologyUniversity Hospital of Southampton NHS Foundation TrustSouthampton, SO16 6YDUK

## Abstract

We report the use of pasireotide in a rare and unusual case of pituitary macroadenoma co-secreting GH, prolactin and ACTH. A 62-year-old Caucasian man presented with impotence. Clinically, he appeared acromegalic and subsequent investigations confirmed GH excess and hyperprolactinaemia. Magnetic resonance imaging (MRI) of pituitary revealed a large pituitary macroadenoma. He underwent trans-sphenoidal surgery and histology confirmed an adenoma with immunohistochemistry positive for ACTH, GH and prolactin. Acromegaly was not cured following surgery and inadequately controlled despite subsequent octreotide therapy. He underwent further debulking pituitary surgery, following which IGF1 levels improved but still high. This time adenoma cells showed immunohistochemistry positivity for ACTH only, following which subsequent investigations confirmed intermittent hypercortisolaemia compatible with pituitary Cushing's disease. We recommended radiotherapy, but in view of the pluripotential nature of the tumour, we proceeded with a trial of s.c. pasireotide therapy on the basis that it may control both his acromegaly and Cushing's disease. After 3 months of pasireotide therapy, his mean GH and IGF1 levels improved significantly, with improvement in his symptoms but intermittent hypercortisolaemia persists. His glycaemic control deteriorated requiring addition of new anti-diabetic medication. MRI imaging showed loss of contrast uptake within the tumour following pasireotide therapy but no change in size. We conclude that our patient has had a partial response to pasireotide therapy. Long-term follow-up studies are needed to establish its safety and efficacy in patients with acromegaly and/or Cushing's disease.

**Learning points:**

- Plurihormonal pituitary adenomas are rare and unusual.
- Patients with pituitary adenomas co-secreting ACTH and GH are more likely to present with acromegaly because GH excess can mask hypercortisolaemia.
- Pasireotide holds potential where conventional somatostatin analogues are not effective in acromegaly due to higher affinity for somatostatin receptor subtypes 1, 2, 3 and 5.
- Significant deterioration in glycaemic control remains a concern in the use of pasireotide.
- Currently, long-term safety and efficacy of pasireotide in patients with acromegaly and/or Cushing's disease are not fully clear.

## Background

Pituitary adenomas co-secreting adrenocorticotrophic hormone (ACTH) and growth hormone (GH) are extremely rare, although GH-secreting adenomas co-secrete prolactin in nearly 30–50% of cases (1). A careful English medical literature search revealed 15 possible cases of pituitary adenomas secreting both ACTH and GH (2) (3) (4) (5) (6) (7) (8). This cannot be confirmed in five of these cases, due to unavailability of modern immunohistochemistry techniques (2) (3). Pasireotide, a recently developed cyclohexapeptide somatostatin analogue (SSA) that binds and activates somatostatin receptor (SSTR) subtypes 1, 2, 3 and 5, is the first drug to be approved specifically for the treatment of Cushing's disease in the European Union (9). Pasireotide has also shown good efficacy in biochemical control of acromegaly, although it is not currently licensed in Europe for this indication (10) (11). We report the first case where pasireotide was used in a patient who had a plurihormonal pituitary adenoma co-secreting ACTH, GH and prolactin.

## Case presentation, investigations and treatment

**Figure 1:**
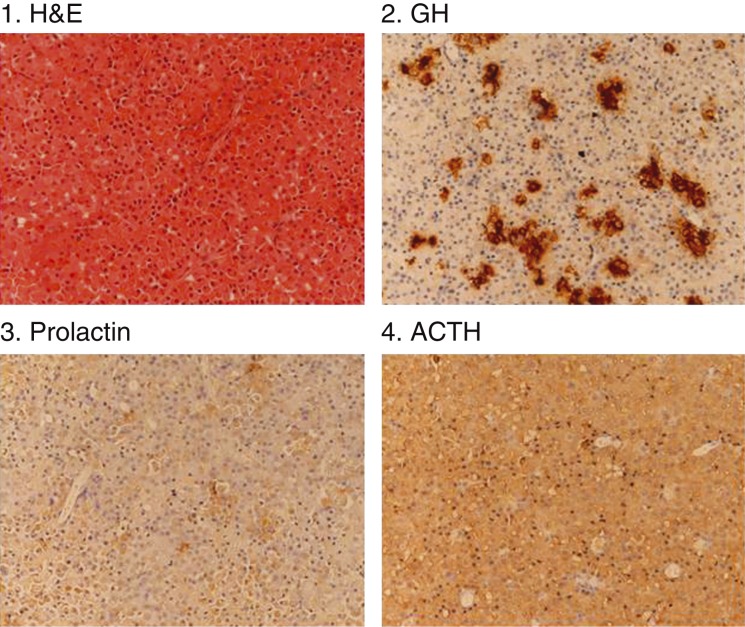
Following first pituitary surgery. Histological examination showed acidophilic adenoma composed of cells with regular rounded nuclei and granular (1) eosinophilic cytoplasm (H&E, magnification ×200). The adenoma cells showed diffuse immunopositivity for (4) ACTH (magnification ×40) and focal but strong reactivity for (2) GH (magnification ×40) and (3) prolactin (magnification ×40).

A 62-year-old Caucasian man with a medical history of type 2 diabetes mellitus (T2DM) for 1 year, hypertension for 10 years and left ventricular hypertrophy presented with impotence and prostatism in 2008. He had the classical appearance of acromegaly. Subsequent investigations showed prolactin 12550 mU/l (55.4–276), random GH 32 μg/l, follicle-stimulating hormone (FSH) 2.3 IU/l (1.3–19.3), luteinizing hormone (LH) 0.2 IU/l (1.2–8.6), testosterone 1.89 nmol/l (6.7–40), thyroid-stimulating hormone (TSH) 0.44 mU/l (0.34–5.6), free thyroxine (T_4_) 11.3 pmol/l (7.5–21.1) and random cortisol 366 nmol/l. Magnetic resonance imaging (MRI) scan of pituitary gland revealed 2 cm macroadenoma invading both cavernous sinuses and petrous portion of internal carotid arteries, more so on the left side, with extension into the suprasellar space but optic chiasm was free from tumour. He was commenced on cabergoline 250 μg twice a week and subsequently underwent subtotal trans-sphenoidal decompression of the pituitary tumour. Complete removal was not achieved due to tumour extent. The adenoma cells showed diffuse immunopositivity for ACTH and focal but strong reactivity for GH and prolactin (Fig. 1). There was no expression of LH, FSH and TSH. The proliferation rate (Ki67 labelling index) was low.

**Figure 2:**
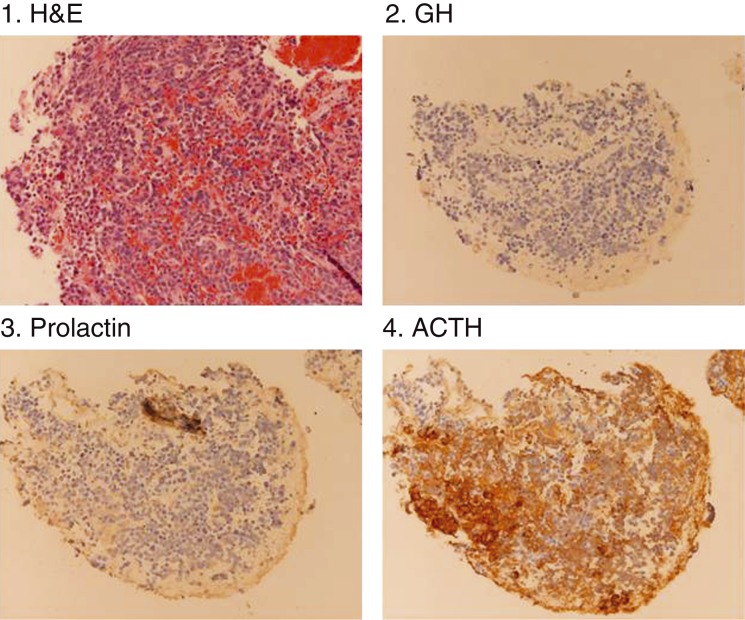
Following second pituitary surgery. Histological examination this time showed adenoma cells with lack of (1) hypereosinophilic cytoplasm (H&E, magnification ×200). Immunohistochemical staining of the adenoma cells surprisingly showed positivity for (4) ACTH only (magnification ×20) and were negative for (2) GH (magnification ×20) and (3) prolactin (magnification ×20).

Biochemical testing confirmed that his acromegaly was not cured post-operatively, insulin-like growth factor 1 (IGF1) 842 ng/ml (75–212). He remained on cabergoline and was commenced on octreotide LAR injection 30 mg once every 4 weeks and testosterone undecanoate injection 1 g every 12 weeks for secondary hypogonadism. Despite this, he had inadequate control of acromegaly with IGF1 771 μg/l (52–282) and mean GH 4.7 μg/l on a five-point GH day curve. Prolactin level was 33 mU/l (55–276). There was a significant residual tumour with extension into the cavernous sinuses with homogenous enhancement on MRI scan of the pituitary. At this stage, he underwent further debulking trans-sphenoidal surgery to remove as much of the sellar component of the tumour in April 2011. Immunohistochemical staining of the adenoma cells surprisingly showed positivity for ACTH only and were negative for GH, prolactin, FSH, LH and TSH (Fig. 2). The proliferation rate (Ki67 labelling index) remained low.

**Figure 3:**
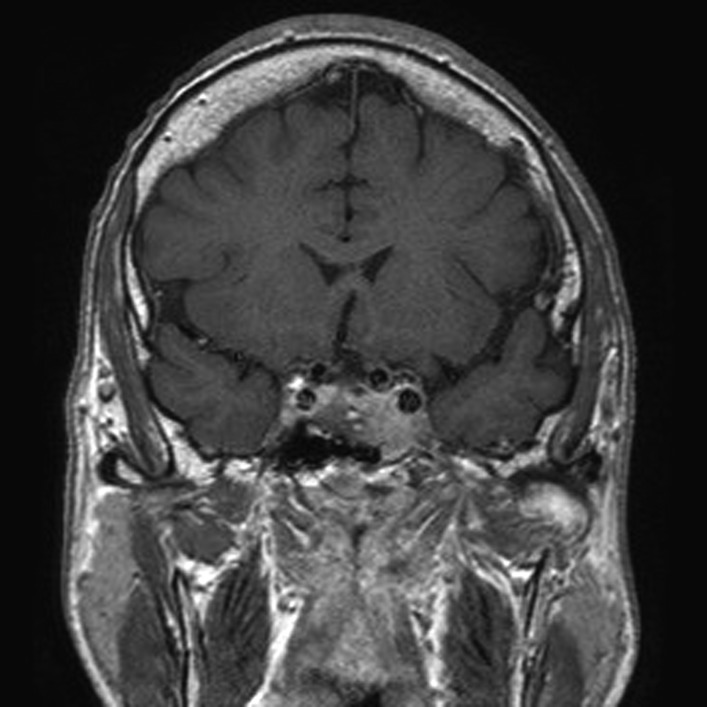
Before pasireotide therapy. MRI scan of pituitary – coronal view – T1 weighted image with gadolinium contrast showing residual pituitary tumour with invasion of cavernous sinus showing homogenous enhancement.

Post-operatively, he reported significant fatigue and weight gain. His thyroid function result (TSH 0.30 mU/l (0.34–5.6) and free T_4_ 9.2 pmol/l (7.5–21.1)) was suggestive of secondary hypothyroidism and he was commenced on l-T_4_ 100 μg daily. In view of the second histology result, he was investigated for possible hypercortisolaemia. Midnight salivary cortisol was significantly elevated at 15.8 and 19.1 nmol/l (<5) on two occasions. There was intermittent hypercortisolaemia with intermittent elevated 24-h urine free cortisol (UFC) 251 and 457 nmol (<280) on two occasions, with failure to suppress on both the 1-mg overnight dexamethasone suppression test (0900 h cortisol 177 nmol/l) and 48-h low-dose dexamethasone suppression test (cortisol 105 nmol/l). His serum ACTH measured on multiple occasions was in the normal range 10, 18 and 29 ng/l (0–40). He continued to have elevated IGF1 of 314 nmol/l (39–242) and mean GH of 2 μg/l on a five-point day curve while on octreotide LAR therapy. We surmised him to have concurrent active acromegaly and pituitary Cushing's disease based on biochemistry and histological findings. MRI scan of the pituitary showed slight reduction in size of the residual pituitary adenoma (Fig. 3).

We recommended radiotherapy, but in view of the pluripotential nature of his pituitary adenoma, we commenced him on a trial of s.c. pasireotide therapy on the basis that pasireotide may potentially treat both his acromegaly and Cushing's disease simultaneously. Octreotide LAR injections were discontinued and he was commenced on pasireotide 0.6 mg s.c. twice daily, started 6 weeks following his last octreotide LAR injection.

## Outcome and follow-up

Within 4 weeks of pasireotide therapy, he reported significant improvement in his general well-being with improved energy, exercise tolerance and mood. After 3 months, there was a significant reduction in mean GH level (0.2 μg/l) and IGF1 normalised at 64 μg/l (39–242). However, intermittent hypercortisolaemia persisted with intermittent elevated 24-h UFC 254, 435 and 316 nmol (<280) on three occasions and elevated midnight salivary cortisols 7.2, 9.4, 8.6 and 11.4 nmol/l (<5) on four occasions. Pasireotide dose was temporarily increased to 0.9 mg twice daily for 4 weeks but he did not tolerate this due to gastrointestinal side effects.

**Figure 4:**
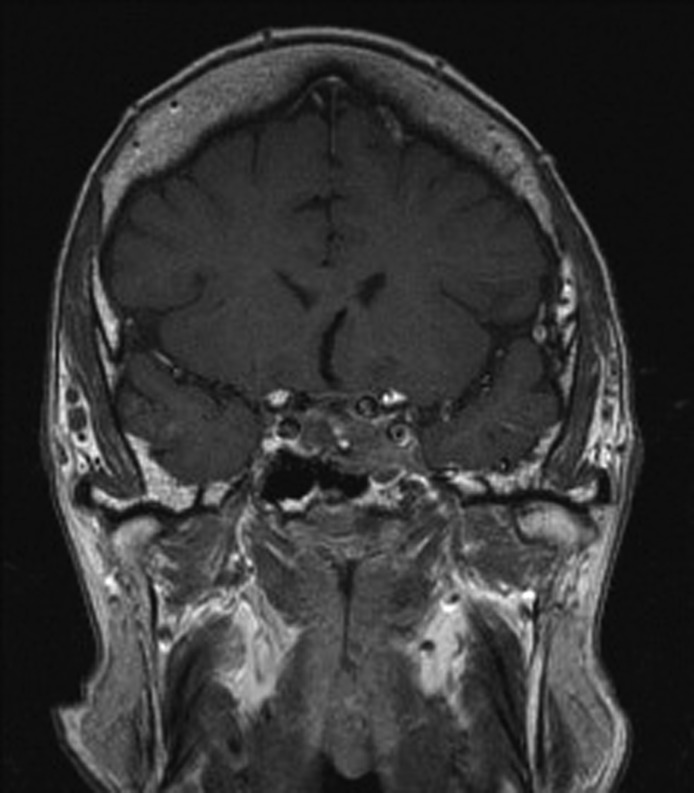
Three months after pasireotide therapy. MRI scan of pituitary – coronal view – T1 weighted image with gadolinium contrast showing residual pituitary tumour with invasion of cavernous sinus showing loss of enhancement.

He developed significant deterioration in glycaemic control and HbA1c increased from 58.5 mmol/mol (7.5%) to 79.2 mmol/mol (9.4%), within 2 months of pasireotide therapy. Following introduction of sitagliptin, this improved to 66 mmol/mol (8.2%). MRI of pituitary gland at 3 months showed change in tumour appearance with loss of contrast enhancement but no significant change in size of the tumour (Fig. 4). He awaits radiotherapy while pasireotide has been continued as bridging therapy.

## Discussion

Plurihormonal pituitary adenomas are different from double adenomas in pituitary gland in that plurihormonal adenomas are single adenomas that produce more than one hormone whereas double adenomas are two morphologically and/or immunohistochemically distinct tumours (12). Plurihormonal adenomas could be either monomorphous (one cell type secreting multiple hormones) or plurimorphous (multiple cell types, each secreting one hormone) (7). We surmise that our case was a plurihormonal pituitary adenoma rather than a double adenoma based on his first post-operative histological and immunohistochemical examination, although clonal analysis and electron microscopic study was not performed. The absence of GH and prolactin staining on the second post-operative specimen could reflect either selection of a particular clone of cells over time or pretreatment with octreotide whose morphological effects are still not fully understood (1) rather than sampling of a different area of the tumour.

Even though our case had biochemical evidence of hypercortisolaemia along with uncontrolled acromegaly, he had predominantly acromegalic features with no florid signs of Cushing's disease and his metabolic complications (T2DM and hypertension) could be attributed to either his GH and/or his cortisol excess. Among the 15 possible cases of coexistent acromegaly and Cushing's disease described in the literature (2) (3) (4) (5) (6) (7) (8), at least nine cases had predominant acromegalic features and only two cases had predominant Cushingoid features, and it was not ascertainable what features predominated in the remaining four cases. There are several theories explaining this, such as production of low-activity high-molecular weight ACTH (8) or biologically inactive ACTH molecules due to impaired PC 1/3 expression in these tumours or impaired intracellular secretory process of ACTH compared with GH (7). Finally, hypercortisolaemia can be masked by GH excess. GH excess inhibits 11β-hydroxysteroid dehydrogenase type 1 that converts inactive cortisone to active cortisol, resulting in reduced tissue exposure to cortisol (13).

Ninety per cent of GH-secreting adenomas express SSTR2 and SSTR5. Conventional SSA's octreotide and lanreotide have high affinity for SSTR2 and modest affinity for SSTR5 and normalise GH levels only in 48–67% of cases (10). In Cushing's disease, ACTH secreting adenomas predominantly express SSTR5 but SSTR2 expression is also seen (9) (14) (15), which may be involved in the hypercortisolaemia seen in our case. On direct comparison with octreotide, pasireotide has 40-, 30- and 5-fold higher affinity for SSTR5, SSTR1 and SSTR3 respectively and twofold lower affinity for SSTR2 (10) and holds potential where conventional SSA's are ineffective such as ACTH secreting adenomas and octreotide-resistant GH-secreting adenomas. Recently, pasireotide has been shown to be effective in decreasing cortisol levels in patients with Cushing's disease in a randomised multicentre 12-month phase 3 study (15) with another recent phase 3 study suggesting superior efficacy compared with octreotide LAR in the management of acromegaly (11). Although the safety profile of pasireotide was similar to that of conventional SSA's in terms of gastrointestinal symptoms and gallstones, higher frequency of hyperglycaemia was observed and is thought to be due to decreased insulin and incretin secretion (15). While our patient's acromegaly improved significantly with decline in GH and IGF1 levels when octreotide was switched to pasireotide, he continued to have intermittent hypercortisolaemia and his glycaemic control deteriorated significantly requiring a new anti-diabetic medication.

In conclusion, our patient has shown partial response to pasireotide therapy. He has been referred for external beam radiotherapy as we felt that that his hypercortisolaemia may worsen with time. In view of improvement in his symptoms and biochemical control of acromegaly, pasireotide has been continued as bridging therapy while awaiting response to radiotherapy. Pasireotide is a promising new treatment option in patients with acromegaly and/or Cushing's disease but long-term safety and efficacy are not entirely clear, and long-term follow-up studies are needed to establish this.

## Patient's perspective

The initial improvement has maintained but fatigue is still a factor in my life and the improvement is not even. Some days I feel quite well but others quite poorly, and this variation can also happen within a day.

## Patient consent

Written informed consent was obtained from the patient for publication of this case report.

## Author contribution statement

R Rajendran was responsible for case description, literature review and writing. S Naik and D D Sandeman were the patient's physicians and were responsible for editing. A B Nasruddin was the patient's physician and was responsible for case description, literature review and editing.
